# AttSec: protein secondary structure prediction by capturing local patterns from attention map

**DOI:** 10.1186/s12859-023-05310-3

**Published:** 2023-05-04

**Authors:** Youjin Kim, Junseok Kwon

**Affiliations:** 1 Department of Artificial Intelligence, Chung-Ang University, Seoul, Republic of Korea; 2 LG AI Research, Seoul, Republic of Korea

**Keywords:** Protein secondary structure, Computational biology, Transformer, 2D Convolution

## Abstract

**Background:**

Protein secondary structures that link simple 1D sequences to complex 3D structures can be used as good features for describing the local properties of protein, but also can serve as key features for predicting the complex 3D structures of protein. Thus, it is very important to accurately predict the secondary structure of the protein, which contains a local structural property assigned by the pattern of hydrogen bonds formed between amino acids. In this study, we accurately predict protein secondary structure by capturing the local patterns of protein. For this objective, we present a novel prediction model, AttSec, based on transformer architecture. In particular, AttSec extracts self-attention maps corresponding to pairwise features between amino acid embeddings and passes them through 2D convolution blocks to capture local patterns. In addition, instead of using additional evolutionary information, it uses protein embedding as an input, which is generated by a language model.

**Results:**

For the ProteinNet DSSP8 dataset, our model showed 11.8% better performance on the entire evaluation datasets compared with other no-evolutionary-information-based models. For the NetSurfP-2.0 DSSP8 dataset, it showed 1.2% better performance on average. There was an average performance improvement of 9.0% for the ProteinNet DSSP3 dataset and an average of 0.7% for the NetSurfP-2.0 DSSP3 dataset.

**Conclusion:**

We accurately predict protein secondary structure by capturing the local patterns of protein. For this objective, we present a novel prediction model, AttSec, based on transformer architecture. Although there was no dramatic accuracy improvement compared with other models, the improvement on DSSP8 was greater than that on DSSP3. This result implies that using our proposed pairwise feature could have a remarkable effect for several challenging tasks that require finely subdivided classification. Github package URL is https://github.com/youjin-DDAI/AttSec.

## Background

Proteins are chains of amino acids, in which approximately 20 kinds of amino acids can make an infinite number of proteins by changing their arrangement. This sequence of amino acids is called the primary structure of the protein (1D sequence). In the human body, proteins are spatially coiled, bent, and folded due to the interaction of amino acids, which induces a specific three-dimensional structure (3D structure). This is called the tertiary structure of protein. Many recent studies aim to predict this tertiary structure because several unique properties of protein can be derived from this structure [1–3]. However, it is very difficult to directly predict the 3D structure from the 1D sequence. To alleviate this difficulty, the secondary structure of protein is predicted, which links the 1D sequence to the 3D structure. Please note that the secondary structures can be intermediate features for the complex 3D structures and used as to represent the local properties of proteins. The secondary structures are typically assigned by the DSSP (Define Secondary Structure of Proteins) algorithm [4, 5]. The DSSP algorithm checks whether there is hydrogen bond for each amino acid pair by identifying the distance between the elements given the 3D coordinate file of the protein. Then, based on the local patterns of these hydrogen bonds, eight types of secondary structure are assigned to amino acids (DSSP8): 3-Helix (G), $\alpha$-Helix (H), 5-Helix(I), hydrogen bonded turn (T), residue in isolated $\beta$-bridge (B), extended strand participates in $\beta$ ladder (E), bend (S), and coil (C). The aforementioned types can be further grouped into three larger classes (DSSP3): helix (H), strand (E), and loop (C). While there are several ways to reduce the 8 types to 3 types, we use general reduction: (G/H/I $\rightarrow$ H, E/B $\rightarrow$ E, S/T/C $\rightarrow$ C).

Due to the lack of data and the difficulty of prediction, conventional methods for secondary structure prediction rarely use only a single sequence and highly rely on additional evolutionary information. For example, Multiple Sequence Alignment (MSA) in [6] and Position-Specific Scoring Matrix (PSSM) in [7] have been generated from other databases and used together with sequence data to predict protein structure. However, while constructing MSA or PSSM for each template sequence requires high effort, it is difficult to expect good performance for proteins with few or no homology sequences. To overcome this, a language model was employed in [8, 9], which has proven performance in the field of natural language processing. If the language model is pretrained with large unlabeled data and finetuned for a downstream task, the model can achieve outstanding performance even if only a small amount of the downstream task data is available. In this context, the embedding of a language model was used in [8, 9] to replace the evolutionary information by showing that the embedding of a language model that was pretrained with a pretext task with large protein sequence data could perform properly in protein-related downstream tasks like protein structure prediction, subcellular localization prediction, and membrane prediction. Inspired by these methods, our model also utilizes the protein embedding of a pretrained language model as an input instead of using the additional evolutionary information. Recently, there have been models that predict protein secondary structure by using language model’s embeddings instead of MSA, such as SPOT-1D-LM [10] and NetsurfP-3.0 [11]. SPOT-1D-LM employs ensemble learning by training three models with the embeddings of two different language models, ProtT5-XL-U50 and ESM-1b. Their models include one LSTM-based model and two 1D CNN-based models. Similarly, NetsurfP-3.0 also uses ESM-1b’s embedding and combines LSTM and 1D CNN to construct model. Both models have the common feature of having network structures that extract features sequentially in addition to using language model embeddings. In contrast, our proposed model, AttSec, takes a different approach to accurately describing the way secondary structures are assigned to each amino acid constituting a protein.

The secondary structure is determined by the patterns of hydrogen bonds, which correspond to pairwise features between amino acids. Then, the patterns of hydrogen bonds correspond to the local patterns of pairwise features between amino acids. To implement the aforementioned hierarchical approach via model design, AttSec extracts the self-attention map corresponding to the pairwise features between amino acid embeddings and passes it through 2D convolutional blocks to detect the local pattern. Thus, AttSec mainly consists of two parts. The first part has multiple layers of the transformer encoder to estimate the self-attention maps. When a secondary structure is assigned, different secondary structures can be assigned depending on how far apart amino acids form hydrogen bonds. Thus, to consider the importance of this relative distance, AttSec constructs a transformer encoder layer using relative position encoding (RPE) instead of conventional absolute position encoding (APE). In the second part, the 2D segment detector detects different patterns of hydrogen bonds from the stack of pairwise features. By using a convolutional kernel with different options per block, we ensure that the model gives robust detection results.

The contributions of our method are as follows.

- We use protein embedding of the language model to replace additional evolutionary information, in which there is no significant drop in performance even for sequences with no or few homology sequences.
- We describe the way that protein secondary structures are assigned by processing sequential features into pairwise features and detecting local patterns based on transformer-based deep learning compared with existing models that simply extract features in a sequential manner.

## Methods

### Dataset

We trained our model using two datasets for efficient comparison with baseline models. One is ProteinNet in [12] and the other is the NetSurfP-2.0 dataset in [13]. The first dataset, ProteinNet, is a benchmark dataset for protein structures and is built from PDB structures that were released as of 2016. ProteinNet provides data with different sequence identity cutoffs applied. Among data, we used a dataset with cutoff of 95% as used in [14]. The number of sequences in this training dataset is 39, 120. However, because the secondary structure data provided by ProteinNet was incomplete and not all data sequences could be assigned secondary structures with the DSSP program, we were able to use 38, 000 data for training. As the validation set, 100 proteins were used, which were same as those provided by [14]. The model that was trained in this way was evaluated using the SPOT-2016, SPOT-2016-HQ, SPOT-2018, SPOT-2018-HQ, and TEST-2018 datasets. These test datasets were also used in [14]. The training dataset, Proteinnet, includes protein structures released up to 2016. They constructed the SPOT-2016 dataset using proteins released between 2016 and 2020. Among them, proteins with an e-value cutoff of less than 0.1 in the hidden Markov model comparison with pre-2016 proteins were all removed. In addition, from the SPOT-2016 dataset, they gathered only the proteins released after 2018 to form SPOT-2018, and those with the HQ suffix were subsets with the resolution constraint applied. Moreover, the TEST-2018 dataset consists of high-resolution proteins released only in 2018, filtered at a 25% identity threshold with pre-2018 proteins. Because the ProteinNet dataset provided only the data for the 8-states DSSP assigned by the DSSP program, an additional reduction process was required to obtain the 3-states DSSP (DSSP3) data. Thus, we made DSSP3 data by converting DSSP8 to DSSP3 according to the general reduction method (G/H/I $\rightarrow$ H, E/B $\rightarrow$ S, S/T/C $\rightarrow$ C). The second dataset, NetSurfP-2.0, provided by [13] can be downloaded simply in CSV format. NetSurfP-2.0 provides 10,792 data samples both in 3-states and 8-states DSSP. For validation of the model trained with this data, we used 646 protein data samples, including CASP12, CB513, and TS115 as in [8]. The model trained with NetSurfP-2.0 was evaluated on NEW364, CASP12, CB513, and TS115. The CASP12, CB513, and TS115 datasets are independent datasets used in [13]. Any protein with a sequence similarity of over 25% to any protein in these three datasets was excluded from the training set, but redundancy among the test datasets was not handled. The NEW364 dataset was created in [8] to complement the limitations of these three test sets. It was constructed by selecting proteins from the PDB with a resolution of 2.5 Å or better and a minimum of 20 amino acids, which were published after 2019. MMSeqs and PISCES were used to remove any proteins with more than 20% similarity to either the training data or the dataset itself.

### Pretrained language model

Protein structure prediction tasks are challenging, because the size of the available dataset is small and there are few proteins whose structures are known. Thus, the inattentive use of complex models can cause overfitting problems. Fortunately, there exist extensive databases of proteins whose 3D structures are not known but whose primary sequences are known. Thus, many conventional methods utilize evolutionary information by finding sequences that are similar to the template sequences in a protein sequence database and putting them together as an input to the model. However, because these methods cannot guarantee performance for proteins having few or no homology proteins, recent methods attempt to extract evolutionary information from the protein sequence database in a different way. In the methods [8, 9], language models are pre-trained using large sequence data through a pretext-task to generate evolutionarily meaningful protein embeddings. As with these methods, we use a pretrained language model called ProtT5-XL-U50 [8] to obtain protein embeddings. ProtT5-XL-U50, which is based on T5 [15], is trained using the BFD dataset [16, 17] and the UniRef50 dataset [18] by performing a denoising task proposed in BERT [19] as a pretext task. This model provides 1024-dimension per token (per amino acids) embeddings given the protein primary sequence as an input. We import the pretrained language model and use the embedding derived through the inference as an input to our model. Please note that there is no additional finetuning for the language model.

**Fig. 1:**
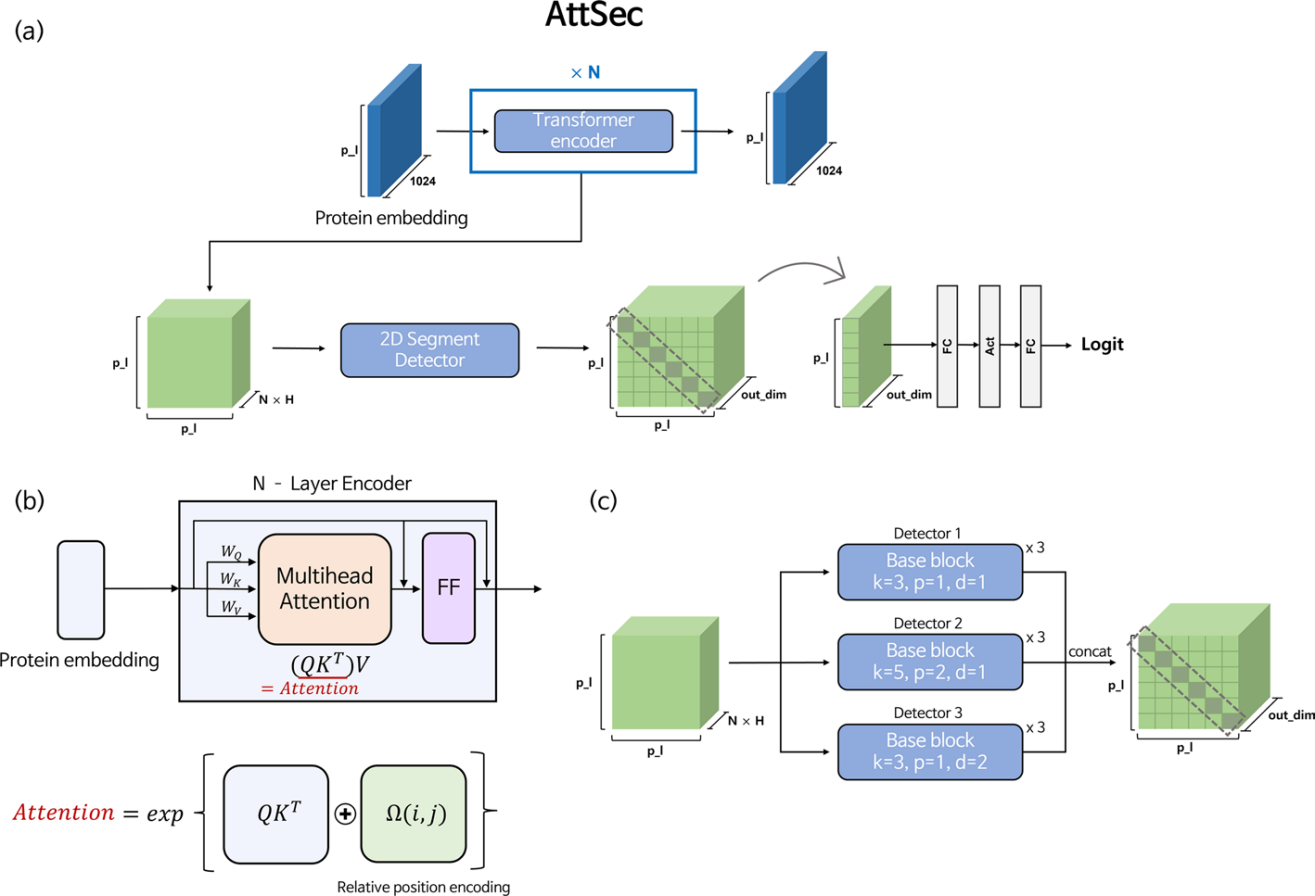
Proposed Secondary Structure Prediction Model (AttSec)** a** Whole network architecture.** b** Transformer encoder.** c** 2D Segement detector

### Proposed secondary structure prediction model

The secondary structure is a local substructure of a protein. To allocate the secondary structure, the DSSP algorithm finds whether there is a hydrogen bond between amino acids and assigns one of eight secondary structures according to the pattern of the hydrogen bonds in the local region. To effectively capture these complex and hierarchical properties, we design the transformer-based deep neural network model in stages. As shown in Fig. 1(a), AttSec obtains attention maps by passing the protein embedding through the multiple transformer encoder layers. The attention map can be stacked as many as the number of heads per encoder layer. Thus, in the case of a protein with a total sequence length of *p*, the shape of the attention map is $p \times p \times (N \times H)$ if it passes through a transformer encoder with *N* layers and *H* heads. Then, this stack of attention maps that corresponds to the pairwise features between amino acids is passed through the 2D segment detector so that the convolutional blocks capture meaningful local patterns. To predict the secondary structure for each token (amino acid), we transform the 2D shape features obtained by the convolutional blocks into 1D shape features. Our model conducts this process in a simple way by extracting only the diagonal elements of the 2D feature. In the 2D segment detector, because several layers of 2D convolution blocks are stacked, considering the receptive field, the diagonal elements of the final feature contain information about the local pattern of pairwise interactions around the target token that the secondary structure wants to know. Finally, these diagonal elements pass through the two fully connected layers to make the final prediction. The whole model consists of two parts: the transformer encoder shown in Fig. 1b and the 2D segment detector shown in Fig. 1c.

#### Transformer encoder layer

For the position encoding of the transformer encoder, we use a variant of relative position encoding (RPE). In vanilla transformers [20], absolute position encoding (APE) is employed to use the sinusoidal function based on the absolute position of the tokens. On the other hand, RPE is implemented based on the relative position of each token when self-attention is calculated, without considering the absolute position of the token. For our task, RPE is more suitable for position encoding than APE, because we regard the self-attention calculated with amino acid (token) embedding pairs as a feature related to hydrogen bonds formed between amino acids. By detecting local patterns from this, the secondary structure can be predicted. Thus, if the position embedding for the relative distance of the amino acid pair is added to the self-attention as a learnable form, it helps to distinguish the different patterns of hydrogen bonds. This is because different secondary structures are assigned depending on the distance between amino acids that form hydrogen bonds. For example, if a pattern in which the *i*-th amino acid forms a hydrogen bond with the $(i+3)$-th amino acid appears in a local area, a 3-turn helix (G) is assigned, but a pattern in which the *i*-th amino acid forms a hydrogen bond with the $(i+4)$-th amino acid appears, a 4-turn helix (H) is assigned. Thus, we utilze RPE as the position encoding to consider the importance of the relative distance between amino acids that form hydrogen bonds. The basic RPE proposed by [15] calculates the relative position and then assigns buckets according to distance. In this study, we modify the vanilla RPE with two changes in the way the buckets are allocated. The first change is to make the relative position bucket symmetric by assigning the same bucket if the relative distance is the same. The second change is that the range to which the bucket is allocated does not increase logarithmically but increases linearly to the specific distance, so that it is more sensitive to the relative position of amino acids.

#### 2D segment detector

The proposed 2D segment detector for detecting local patterns from the stacked self-attentions is composed of 3 detectors, in which each detector is constructed by stacking 3 base blocks. The base block used in our detector has the same structure as the block used in [21]. This base block includes both channel attention and pixel attention, and it enables flexible learning by calculating weights for pixel-wise features and channel-wise features, respectively. The pixel-wise features from [21] can be considered as interaction-wise features in our detector. We extract various features by setting different options for the kernel used for each detector differently to enable the robust detection of local patterns. Conv2D kernels with a size of 3, padding 1, and dilation 1 are used in the first detector, Conv2D kernels with a size of 5, padding 2, and dilation 1 are used in the second detector, and Conv2D kernels with a size 3, padding 1, and dilation 2 are used in third detector. The features that pass through each detector are concatenated in a dimension-wise manner. Because there is no contraction of the feature due to the repeated use of padding in Conv blocks, the shape of the final feature that passs through the 2D segment detector becomes $P \times P \times out\_dim$, as shown in Fig. 1c.

### Training detail

Protein sequence data has a variable length for each sequence. In addition, because we transform sequential features into pairwise features during training, there is a large difference between the amount of computation and memory usage according to the length of the input sequences. Thus, it is necessary to process long sequences for stable training. Rather than cutting the sequence to a certain length during the preprocessing, we randomly crop it every epoch to enable efficient training while obtaining an augmentation effect. For training, cross entropy loss was used, the batch size was set to 2, and the number of epochs was set to 10. As a scheduler, cosineAnnealingLR was used to prevent the model from becoming trapped in local minima. The specific details of the model are as follows: the transformer encoder has 3 layers and 8 heads, resulting in a total dimension of 24 for the constructed attention map. The channel size of the convolution blocks used in the segment detector is set to 64 for all layers.

## Results and discussion

### Performance comparison

We used two datasets for training and compared the performance between different models. The model trained with ProteinNet was compared with PSIPRED [22], SPIDER3 [23], ProteinUnet [24], SPOT-1d single [14] that used only a single sequence as an input, and SPOT-1D [25] that used additional evolutionary information. Additionally, SPOT-1D-LM, which also uses language model embeddings similar to our method, was compared separately as it can only perform inference on sequences with a length of 1024 or less. These models were evaluated on the SPOT-2016 (1473 proteins), SPOT-2016-HQ (295 proteins), SPOT-2018 (548 proteins), SPOT-2018-HQ (125 proteins), and TEST-2018 (250 proteins) datasets. The model trained with NetSurfP-2.0 was compared with DeepProtVec, DeepSeqVec [26], ESM-1b, ProtT5-XL-U50, the ProtT5-XXL-U50 and NetsurfP-3.0 that used the embedding of the language model as a model input, and the NetSurfP-2.0 that used additional evolutionary information. These models were evaluated using CASP12-FM (20 proteins), NEW364 (364 proteins), CB513 (511 proteins), and TS115 (115 proteins) datasets. The performance of the aforementioned models was evaluated in terms of accuracy for all datasets.

**Table 1:** Average prediction accuracy of models trained with NetsurfP-2.0 DSSP8 dataset

Model	CASP12	NEW364	CB513	TS115
DeepProtVec	49.7	53.3	48.9	54.4
DeepSeqVec	61.0	64.8	62.7	67.2
ESM-1b	66.0	71.3	70.2	73.4
ProtT5-XL-U50	68.9	74.5	74.6	77.0
ProtT5-XXL-U50	68.1	72.5	71.6	75.1
NetsurfP-3.0	66.4	72.9	72.0	75.7
NetsurfP-2.0 (profile)	70.3	73.9	72.3	75.0
**AttSec(ours)**	**70.6**	**75.5**	**75.2**	**78.5**

The best results were written in boldface

**Table 2:** Average prediction accuracy of models trained with Proteinnet DSSP8 dataset

Model	TEST2018	SPOT-2016	SPOT-2016-HQ	SPOT-2018	SPOT-2018-HQ
PSIPRED-Single	–	–	–	–	–
SPIDER3-Single	59.8	58.9	59.9	57.4	58.0
ProteinUnet	60.3	–	–	-	–
SPOT-1D-Single	62.2	61.4	61.6	60.1	60.0
SPOT-1D (profile)	75.4	69.3	71.7	67.4	70.5
**AttSec(ours) **	**77.4**	**70.4**	**73.0**	**71.2**	**72.3**
SPOT-1D-Single (less than 1024)	65.5	64.1	65.8	64.6	64.8
SPOT-1D-LM (less than 1024)	**77.5**	70.0	72.6	70.9	72.3
**AttSec(ours) (less than 1024)**	77.4	**70.5**	**73.0**	**71.4**	**72.4**

The best results were written in boldface

Tables 1 and 2 show the comparison results of the 8-states secondary structure (DSSP8). The profile indicates that the corresponding model uses additional evolutionary information as an input. The best results are written in boldface. The accuracy of the dataset was obtained by averaging the accuracy of protein sequences in the dataset. In Table 1, we evaluated the models trained with the NetSurfP-2.0 dataset on four datasets. As shown in the table, AttSec exhibited state-of-the-art performance across all datasets and even outperformed the profile-based model that used additional evolutionary information. Our model outperformed the second best performing model by a margin of 0.3 on the CASP12 dataset, 1.0 on the NEW364 dataset, 0.6 on the CB513 dataset and 1.5 on TS115 dataset. In Table 2, we evaluated the models trained with the ProteinNet dataset on five datasets. Similar to Table 1, AttSec achieved the best performance and even surpassed the profile-based model on all datasets. The profile-based model, SPOT-1D, showed the second highest performance with an accuracy difference of 2.0 on the TEST 2018 dataset, 1.1 on the SPOT-2016 dataset, 1.3 on the SPOT-2016-HQ dataset, 3.8 on the SPOT-2018 dataset, and 1.8 points on the SPOT-2018-HQ from AttSec. It is noteworthy that AttSec surpassed the profile-based model by a quite large difference of 3.8 points on the SPOT-2018 dataset. According to [14], the SPOT-2018 dataset has an average of 4.38 effective homology sequences, which is the smallest among the five evaluation datasets. Thus, because AttSec showed the largest performance difference from the profile-based model on this dataset, our model can considerably outperform the profile-based model, especially for protein sequences with few homologous sequences. In addition, when the datasets were reconstructed by excluding sequences with lengths over 1024, our model achieved the best performance in all datasets except for the TEST2018 dataset.

**Table 3:** Average prediction accuracy of models trained with NetsurfP-2.0 DSSP3 dataset

Model	CASP12	NEW364	CB513	TS115
DeepProtVec	62.9	64.7	63.7	66.5
DeepSeqVec	73.0	76.0	77.0	79.0
ESM-1b	76.9	82.6	83.9	84.8
ProtT5-XL-U50	80.1	84.5	86.2	86.6
ProtT5-XXL-U50	79.2	83.3	84.6	85.6
NetsurfP-3.0	77.8	83.3	85.0	85.9
NetsurfP-2.0 (profile)	**82.0**	84.3	85.4	85.7
**AttSec(ours)**	80.8	**85.2**	**86.5**	**87.5**

The best results were written in boldface

**Table 4:** Average prediction accuracy of models trained with Proteinnet DSSP3 dataset

Model	TEST2018	SPOT-2016	SPOT-2016-HQ	SPOT-2018	SPOT-2018-HQ
PSIPRED-Single	68.9	70.3	69.5	68.0	68.0
SPIDER3-Single	72.6	72.0	72.2	71.3	70.8
ProteinUnet	72.6	–	–	–	–
SPOT-1D-Single	74.3	74.3	73.7	73.7	72.1
SPOT-1D (profile)	86.2	**81.7**	**83.1**	80.4	**82.0**
**AttSec(ours) **	**86.6**	81.6	81.9	**81.5**	81.3
SPOT-1D-Single (less than 1024)	76.5	76.0	75.9	75.9	75.0
SPOT-1D-LM (less than 1024)	**86.7**	81.3	81.8	81.4	**81.6**
**AttSec(ours) (less than 1024)**	86.5	**81.7**	**81.9**	**81.6**	81.4

The best results were written in boldface

Tables 3 and 4 compare the 3-states secondary structure (DSSP3) by reducing DSSP8. Table 3 shows the comparison of the models trained on the NetSurfP-2.0 dataset. As shown in the table, AttSec showed the best performance on three datasets except for the CASP12-FM dataset. AttSec lagged behind the best model by 1.2 points on the CASP12-FM dataset, but improved by 0.7 points on the NEW364 dataset, by 0.3 points on the CB513 dataset, and by 0.9 points on the TS115 dataset. Table 4 includes the evaluation results for training with ProteinNet. AttSec showed comparable performance to the profile-based model. There was a performance difference of $+0.4$ on the TEST2018 dataset, $-0.1$ on the SPOT-2016 dataset, $-1.2$ in the SPOT-2016-HQ dataset, $+0.9$ on the SPOT-2018 dataset, and $-0.7$ points on the SPOT-2018-HQ dataset. Although AttSec outperformed the profile-based model on two out of five datasets, it outperformed the rest of the single sequence-based models on all datasets. On datasets consisting only of short sequences, our model showed similar performance to SPOT-1D-LM. Please note that we was not able to perform inference on all the models (methods), and for some, We had to rely on the performance tables from the reference papers. Therefore, it is difficult to calculate the accuracy for each individual data point that makes up the dataset.

**Table 5:** Performance metric for models trained on Proteinnet DSSP3

Model	Precision	Recall	f1-score	Matthews Corr
SPOT-1D-Single	0.74	0.74	0.74	0.57
SPOT-1D-LM	0.81	0.80	0.80	0.68
AttSec(ours)	0.81	0.81	0.81	0.68

**Table 6:** Performance metric for models trained on NetsurfP-2.0 DSSP3

Model	Precision	Recall	f1-score	Matthews Corr
ProtT5-XL-U50	0.85	0.85	0.85	0.77
NetsurfP-3.0	0.85	0.83	0.84	0.76
AttSec(ours)	0.86	0.86	0.86	0.78

**Table 7:** Performance metric for models trained on Proteinnet DSSP8

Model	Precision	Recall	f1-score	Matthews Corr
SPOT-1D-Single	0.58	0.62	0.58	0.47
SPOT-1D-LM	0.67	0.69	0.67	0.58
AttSec(ours)	0.67	0.70	0.67	0.59

**Table 8:** Performance metric for models trained on NetsurfP-2.0 DSSP8

Model	Precision	Recall	f1-score	Matthews Corr
ProtT5-XL-U50	0.73	0.74	0.72	0.66
NetsurfP-3.0	0.71	0.72	0.70	0.64
AttSec(ours)	0.74	0.75	0.73	0.68

We also provided reports on the precision, recall, F1 score and Matthews Correlation Coefficient on the evaluation datasets in Tables 5, 6, 7, and 8, which allowed for a more comprehensive evaluation of the models’ performance beyond accuracy. Table 5 compares our model with the two best-performing single sequence-based models, SPOT-1D-Single and SPOT-1D-LM, trained on the Proteinnet dataset. To simplify the report, we calculated all metrics on a combined dataset of SPOT-2016, SPOT-2016-HQ, SPOT-2018, SPOT-2018-HQ, and TEST2018. As shown in the table, our model achieved the best performance. Table 6 compares our model with the two best-performing single sequence-based models, ProtT5-XL-U50 and NetsurfP-3.0, trained on the NetsurfP-2.0 dataset. We calculated precision, recall, F1-score and Matthews Correlation Coefficient on the evaluation sets of CASP12, NEW364, CB513, and TS115, and our model outperformed the other models in all metrics. Tables 7 and 8 also demonstrate that our method surpasses methods in terms of the precision, recall, F1 score and Matthews Correlation Coefficient. Overall, it can be seen that there are greater performance differences on DSSP8 than on DSSP3, which can be interpreted to mean that AttSec is specialized in capturing and classifying fine-grained differences between protein secondary structures.

**Fig. 2:**
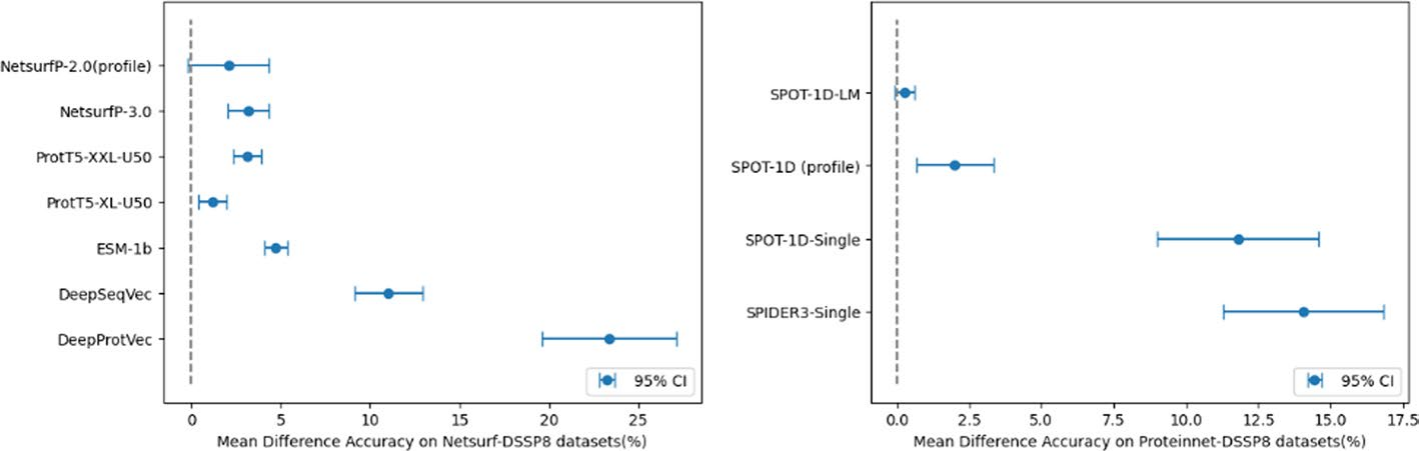
(Left) Mean difference accuracy on NetsurP-DSSP8 evaluation datasets. (right) Mean difference accuracy on Proteinnet-DSSP8 evaluation datasets

**Fig. 3:**
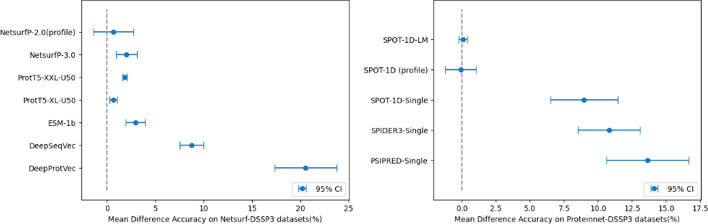
(Left) Mean difference accuracy on NetsurP-DSSP3 evaluation datasets. (right) Mean difference accuracy on Proteinnet-DSSP3 evaluation datasets

To demonstrate that our model exhibits superior performance across the entire dataset in a statistically significant manner, we provide the graphs in Fig. 2. The dot in the middle of each report represents the mean difference value, which indicates the average accuracy difference between our model and each model across all datasets, and a 95% confidence interval is also displayed. As all mean difference values are positive, indicating a higher accuracy for our model compared to other models, and all confidence intervals except for the profile-based NetsurfP-2.0 min confidence interval are also positive, we can conclude that our AttSec model demonstrates significantly higher accuracy compared to other models including SPOT-1D-LM. We also report graphs for models trained with DSSP3 datasets in Fig. 3.

### Ablation study

We conducted three ablation studies. The first study compared the performance of different position encoding methods, the second study compared the performance of models with varying structures, and the third study analyzed the impact of each position encoding on the model’s complexity.

#### Position encoding ablation study

To consider the importance of the relative distance between amino acids that form hydrogen bonds, we used the modified learnable RPE as a position encoding in the transformer encoder layer. In this ablation study, we try to show the effect of modified RPE. For this, we compared our model with three models: a model that does not use any position encoding, a model that applies only APE to the input protein embedding before entering the transformer encoder, and a model that uses both APE and RPE. When APE was used, the max length had to be set during training and the model did not work for sequences longer than that value during inference. Thus, sequences with a length longer than 1024 were removed from each evaluation dataset in this ablation study.

**Table 9:** Comparison of position encoding with NetsurfP-2.0 DSSP8 dataset

Position encoding	CASP12	NEW364	CB513	TS115
RPE (this work)	70.6	75.6	75.2	78.5
No	70.6	75.5	75.2	78.3
APE	67.6	73.0	70.3	75.6
RPE + APE	68.0	73.1	70.6	75.8

**Table 10:** Comparison of position encodings with Proteinnet DSSP8 dataset

Position encoding	TEST2018	SPOT-2016	SPOT-2016-HQ	SPOT-2018	SPOT-2018-HQ
RPE (this work)	77.4	70.5	73.0	71.4	72.4
No	77.1	70.4	72.5	71.3	71.8
APE	75.8	69.4	71.4	70.1	70.7
RPE + APE	74.6	68.8	70.7	69.4	69.8

**Table 11:** Comparison of structures with Proteinnet DSSP8 dataset

Methods	TEST2018	SPOT-2016	SPOT-2016-HQ	SPOT-2018	SPOT-2018-HQ
AttSec(this work)	77.4	70.5	73.0	71.4	72.4
Transformer only	76.9	70.2	72.4	71.1	71.8
LM’s attention	75.3	68.7	70.7	69.4	70.1

**Table 12:** Comparison of structures with NetsurfP-2.0 DSSP8 dataset

Methods	CASP12	NEW364	CB513	TS115
AttSec(this work)	70.6	75.6	75.2	78.5
Transformer only	69.6	74.5	73.3	76.9
LM’s attention map	68.0	73.7	72.1	76.5

Table 9 shows the experimental results of the compared models trained with the NetSurfP-2.0 DSSP8 dataset on four evaluation sets. Table 10 compares the models trained with the ProteinNet DSSP8 dataset on five sets. In both datasets, it can be seen that the model using only RPE exhibits the highest performance, followed by the model without any position encoding. Because the performance is somewhat degraded when APE is added, it can be seen that when extracting self-attention as pairwise features between amino acids, the absolute position of amino acids degrades the pairwise features. The accuracy difference between the model without any position encoding and the model with RPE ranged from 0 to 0.6, which was not large, but the model with RPE always dominated, showing consistency. Because the performance improvement over the model without any position encoding is greater in the ProteinNet dataset, which has more than three times more sequences than NetSurfP-2.0, detecting local patterns from complex pairwise features to which the modified RPE is applied induces greater effect, as there are more data.

#### Model structure ablation study

To demonstrate the effectiveness of the proposed model architecture, we trained and evaluated models with modified structures. The first structure is a model that predicts the secondary structure using only a transformer encoder without a 2D segment detector composed of CNN. The second model is a model that predicts the secondary structure using the attention map of the language model (ProtT5-XL-U50) as an input to the 2D segment detector, without a transformer encoder layer. The performance summary of the two models is presented in Tables 11, 12, 13, and 14. Our model, AttSec, which incorporates a 2D segment detector into the attention maps of the Transformer, performed the best on all datasets. The next best performing model was the Transformer-only model, followed by the model that incorporated a 2D segment detector into the attention maps of the Language model, which had the lowest performance. From these experiments, we can conclude that our novel model architecture, which integrates a 2D segment detector that captures spatial features from meaningful pairwise features extracted in the form of attention maps from the Transformer, was effective in performing this task.

**Table 13:** Comparison of structures with Proteinnet DSSP3 dataset

Methods	TEST2018	SPOT-2016	SPOT-2016-HQ	SPOT-2018	SPOT-2018-HQ
AttSec(this work)	86.6	81.6	81.9	81.5	81.3
Transformer only	86.0	81.4	81.6	81.3	80.9
LM’s attention	85.2	80.1	80.4	80.0	80.0

**Table 14:** Comparison of structures with NetsurfP-2.0 DSSP3 dataset

Methods	CASP12	NEW364	CB513	TS115
AttSec(this work)	80.8	85.2	86.5	87.5
Transformer only	80.3	84.9	85.9	87.0
LM’s attention map	78.3	83.6	84.4	86.2

**Table 15:** Complexity comparison

Positional encoding	Additional parameters	Inference time(s)
RPE (this work)	128	81
No	0	79
APE	0	81

#### Model complexity ablation study

In Table 15, we compared the impact of each position encoding on the model’s complexity by presenting the number of additional trainable parameters and the inference time on the SPOT-1D ($<1024$) dataset, which consists of 1457 protein sequences. Based on the table, it can be seen that adding positional encodings to the Transformer has minimal impact on the overall complexity of the model. Therefore, it is reasonable to use Relative positional encoding in our model, as it provides consistent performance improvements of up to 0.6 by adding only 128 learnable parameters.

## Discussion

We adopted two different training datasets, producing two different models that are used for comparison against different state-of-the-art methods. By adopting two different training datasets and producing two different models, we provided the opportunity to compare our models against state-of-the-art methods trained on different datasets. This approach also allowed us to use other models that have been compared in other papers for inference without requiring additional training. In some cases, the training codes of some models were not publicly available, making it difficult to compare performance accurately. Furthermore, our approach enabled us to evaluate the performance of our models in the same environment as the datasets used in previous research, ensuring a fair comparison. Additionally, using previously validated datasets also increased the reliability of our results.

Recent protein structure prediction models such as AlphaFold2, ESMFold, and RosettaFold have shown remarkable performance. However, these state-of-the-art models primarily focus on predicting the tertiary structure of proteins, operating by directly predicting three-dimensional coordinates. In contrast, our study is centered on predicting the secondary structure of proteins. Considering these differences, we did not perform a direct comparison with models like AlphaFold2, ESMFold, and RosettaFold. These models do not have separate branches specifically designed for predicting secondary structure. Instead, we compared the proposed method to other approaches specialized in secondary structure prediction, considering the differences in the primary objectives of our study and those of the aforementioned models.

## Conclusion

In our study, we used pairwise features that were processed from sequential feature, which can be considered indirect features. Although there was no dramatic accuracy improvement compared with other models, the improvement on DSSP8 was greater than that on DSSP3. This result implies that using our proposed pairwise feature could have a remarkable effect for several challenging tasks that require finely subdivided classification. We also solved the problem of not being able to use a complex-large model due to the lack of protein structure data by using embeddings of language models pre-trained on a vast protein sequence database. In future works, we can look forward to approaches such as extracting more effective pairwise features from elaborately designed models by utilizing embeddings of the language model and adding these pairwise features to existing sequential features to derive significant performance improvements.

## Data Availability

All datasets and the model proposed in this study are available in the GitHub repository. https://github.com/youjin-DDAI/AttSec

## Abbreviations

DSSP: Define secondary structure of proteins
DSSP8: 8-States-DSSP
DSSP3: 3-States-DSSP
MSA: Multiple sequence alignment
PSSM: Position-specifif scoring matrix
PE: Position encoding
APE: Absolute position encoding
RPE: Relative position encoding
